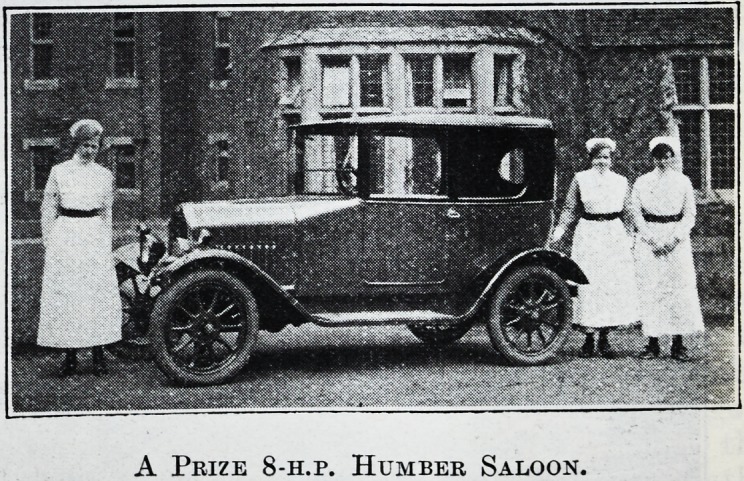# The London School of Hygiene

**Published:** 1924-07

**Authors:** 


### THE LONDON SCHOOL OF HYGIENE.

### WHAT IT WILL DO.

Announcement is made of the appointment of the Governing Body and of the permanent Committee of Management of the London School of Hygiene and Tropical Medicine. At the first meeting of the Governors, when thirty-two members were present, Mr. H. J. Waring was elected Chairman of the Court. The Board of Management is constituted as follows :—Sir Alfred Mond, Sir George Newman, and Sir Walter Fletcher, appointed by the Minister of Health; Sir E. Cooper Perry and Mr. H. J. Waring, appointed by the Senate of the University of London; Captain Sir Arthur Clarke, appointed by the Seamen's Hospital Society; Sir Humphry Rolleston, Colonel H. P. Barrow, and Dr. John Robertson, appointed by the Court of Governors. This School, it will be remembered, is being endowed by the Rockefeller trustees, who have made a munificent gift to the British Government for the establishment of the School, which has yet to be built.

### Combining Education and Research.

Education and research are to be combined in the new School. It is to be established in London, will co-ordinate all the ordinary instruction in public health given in the metropolis, and will absorb the London School of Tropical Medicine. This, however, is only part of its functions, for the scope of public health training is to be extended. There will be courses not only of chemistry but of bio-chemistry and of physiology as applied to hygiene, not only of bacteriology but of immunology, which should be taught in a practical manner, full use being made of laboratories where sera are produced on a big scale. It is very necessary that in the future the State should have at its disposal men thoroughly versed both in the principles and practice of immunology, if only because it is certain sooner or later to exercise control over the manufacture of sera, so far at least as questions of standardisation go. In this direction we are being outstripped by the United States and other countries.

### Training for the Tropics.

At present epidemiology forms a very minor section of public health training. In the new School of Hygiene it is intended to give it a foremost place, and it will be linked with the study of vital statistics and of climatology. From the imperial standpoint, the most significant development is the absorption of the London School of Tropical Medicine, but it is to be hoped that, despite such absorption, the Mansonian tradition will be fully maintained. The union will entail the teaching of the hygiene of the tropics alongside that of the homeland. This is as it should be, for it is merely in detail and in application that the two differ, and each has useful lessons for the other. Opportunity should be taken to train not only Medical Officers of Health, but Sanitary Inspectors destined for work in the tropics (unless instruction for the latter can be furnished by the Royal Sanitary Institute).

### A Great Opportunity.

It would be an excellent thing to have attached to the School of Hygiene a small band of advisers whose duty it would be to go abroad at intervals and to help those responsible for medical and sanitary work in all parts of the British tropics. It is work of this kind which would make the School truly imperial in nature. The great graphic museum built up in connection with the Wellcome Bureau of Scientific Research might be placed in the School and arrangements made to aid research workers coming from abroad, especially from the tropics. If the School is conceived in a parochial spirit and proceeds merely on limited lines a wonderful chance will be lost. English public health, great and worthy though it be, the founder and leader of all modern hygiene, is but the centre of a vast organisation. This organisation, however, is loosely knit. The association of the centre with the rest is rather historical than practical save in a few special directions. Hygiene in some of our overseas possessions is still more or less rudimentary, and moreover, in many of them there are problems which have no parallel in this country. But, the governing principles being the same, there is urgent need for some co-ordinating centre, and that centre should be the new School.

### The Historical Perspective.

Moreover, the history of public health should be taught at the School. The importance of the historical perspective in the study of hygiene must be recognised. The present generation of citizens in this country take their clean streets, their pure water, their cleanly and effective sewage disposal, their conveniences and public health privileges as a matter of course. They do not realise that these things have been earned, and hardly earned, by the pioneers; they never imagine that if the machinery were suddenly to stop they would be face to face with tragedy in less than a month. Yet Russia could have taught them the lesson not long ago.

---

### A FIRM'S GENEROSITY.

A Skill Competition in aid of the Coventry and Warwickshire Hospital has just been organised. The first prize, of which we print a photograph, is

A Prize 8-h.p. Humber Saloon.

an 8-h.p. Humber Saloon Car, given by Humber, Ltd., the well-known Coventry firm. It is expected that this most useful institution will benefit greatly by the competition.

## Figures and Tables

**Figure f1:**